# Haemophagocytic Lymphohistiocytosis Presenting With a Renal Infarct of Unclear Aetiology and Persistent Fever: A Diagnostic Dilemma

**DOI:** 10.7759/cureus.119606

**Published:** 2026-11-13

**Authors:** Yasmin Barud

**Affiliations:** 1 Medicine and Surgery, Luton and Dunstable University Hospital, Luton, GBR

**Keywords:** acute renal infarction, diagnostic delay, haemophagocytic lymphohistiocytosis (hlh), h-score, pyrexia of unknown origin

## Abstract

Haemophagocytic lymphohistiocytosis (HLH) is a rare and potentially fatal hyperinflammatory syndrome characterised by excessive immune activation and progressive multisystem dysfunction. Diagnosis in adults can be challenging due to overlap with infection and malignancy.

This case is based on a gentleman in his mid-30s who presented with acute right-sided flank pain and a right renal infarction. From the second day of admission, he developed persistent pyrexia despite antipyretic therapy and broad-spectrum antibiotics. Over the following two weeks, he developed anaemia, thrombocytopenia, marked hyperferritinaemia, and hypertriglyceridaemia. An H-score of 220 supported the diagnosis of HLH, and his case met 5/8 of the HLH-2004 criteria. Thrombophilia, antiphospholipid, and vasculitis testing were negative; haematology considered the infarct likely arterial, although no cause was identified. A blood culture grew gram-positive cocci in clusters; repeat cultures following readmission were negative after treatment was commenced. Bone marrow examination showed no malignancy or convincing haemophagocytosis.

Following multidisciplinary review, treatment with intravenous methylprednisolone and anakinra was associated with clinical and biochemical improvement. This case highlights the need to consider HLH in patients with persistent fever, falling blood counts, and markedly elevated ferritin despite treatment for an alternative acute diagnosis.

## Introduction

Haemophagocytic lymphohistiocytosis (HLH) can manifest across many age groups and tends to be classified as primary, associated with genetic abnormalities in immune regulation, or secondary, triggered by infections, malignancies, and autoimmune conditions [1]. Diagnosing HLH in adults remains challenging due to its non-specific clinical presentation and considerable overlap with conditions such as sepsis and haematological malignancies [2]. Common clinical and biochemical features include persistent pyrexia, cytopenias, hyperferritinaemia, and hypertriglyceridaemia, which may develop progressively throughout the disease course [3]. Given the substantial mortality associated with untreated HLH, early recognition and timely initiation of treatment remains essential [3].

This case report highlights the diagnostic challenges of recognising HLH in the presence of an alternative acute diagnosis, emphasising the importance of maintaining clinical suspicion when persistent fever and progressive haematological abnormalities remain unexplained.

## Case presentation

A gentleman in his mid-30s presented to the emergency department with sudden-onset right-sided flank pain, multiple vomiting episodes, and polyuria. His known background was limited to type 2 diabetes managed with metformin. A week prior to admission, he had visited his general practitioner (GP) with coryzal symptoms and was treated with amoxicillin. On examination, he was extremely tender in the right flank and right iliac fossa. Initial blood results showed a mildly raised white cell count of 11.7 × 10⁹/L and CRP of 8.7 (unit not specified in the original report). CT of the abdomen and pelvis demonstrated an acute right renal infarction affecting up to 70% of the renal parenchyma (Figure 1). From day 2 onwards, he developed persistent episodes of fever despite antipyretics, repeated courses of antibiotics, and microbiology input.

**Figure 1:**
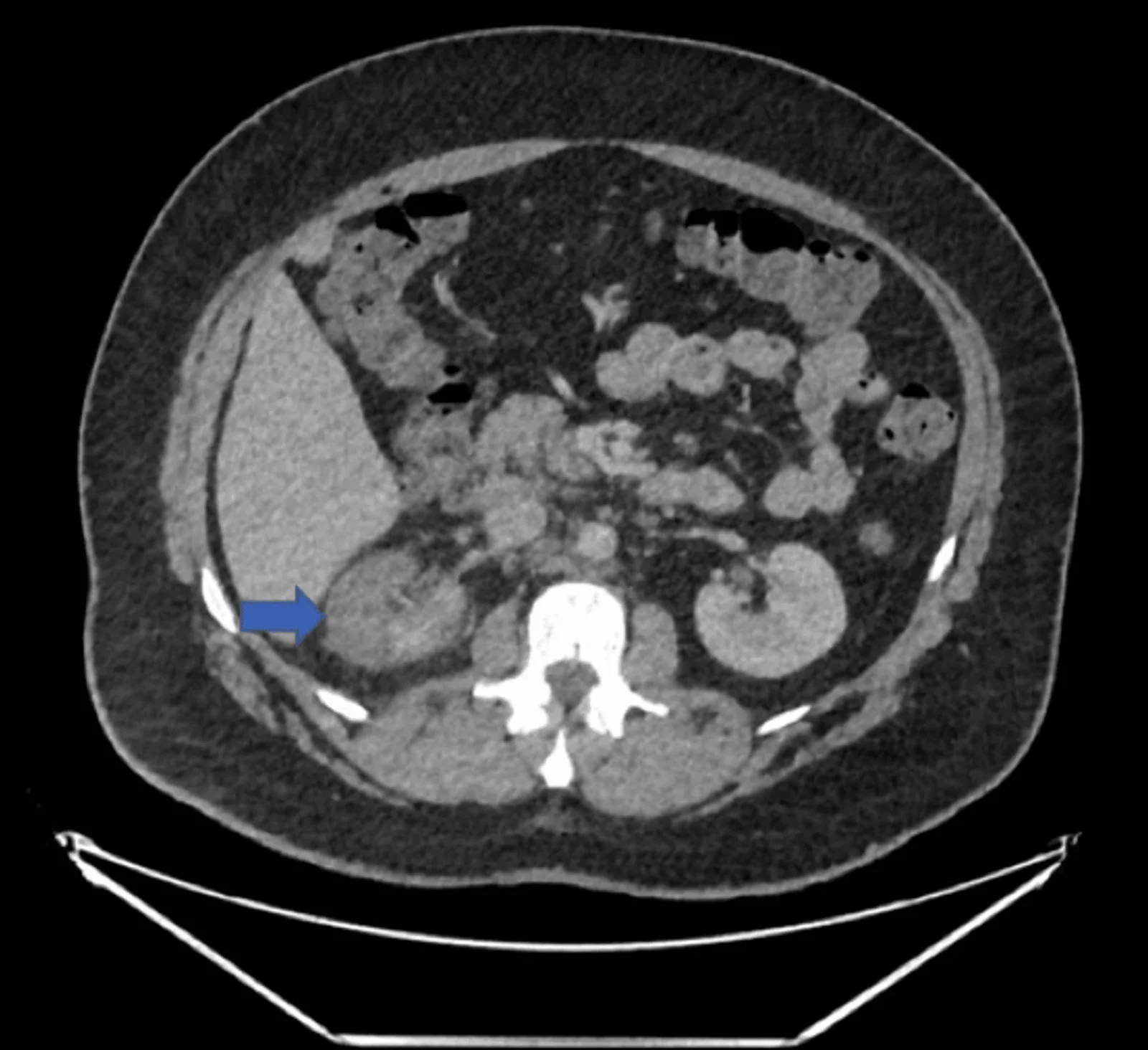
CT (anteroposterior) findings of right renal infarction affecting up to 70% of the renal parenchyma.

Further investigations into the renal infarction included thrombophilia, anti-phospholipid, and vasculitis screens, all of which were negative. ECG demonstrated normal sinus rhythm. Two transthoracic echocardiograms showed no evidence of infective endocarditis or valvular lesions. Following haematology input, the infarction was considered likely to represent an arterial event, although no clear underlying cause was identified following testing.

From day 2, the patient developed persistent temperatures reaching 39°C despite antipyretics and successive antimicrobial regimens. Initial blood and urine cultures and other infection investigations did not identify a source; chest radiography and echocardiography were unrevealing. Piperacillin-tazobactam was followed by teicoplanin and then meropenem; a maculopapular rash prompted discontinuation of meropenem and use of ciprofloxacin following microbiology advice. Tuberculosis, cytomegalovirus, and Epstein-Barr virus investigations were negative. CT of the thorax, abdomen, and pelvis showed mildly enlarged mediastinal lymph nodes and the previously identified infarct; PET-CT did not demonstrate a clear malignant focus.

HLH-specific blood tests were not undertaken until approximately day 12 of admission. Ferritin was initially 6,345 µg/L, rising to a peak of 28,904 µg/L; triglycerides were 8.0 mmol/L. Progressive anaemia and thrombocytopenia developed. For the itemised H-score, the two affected lineages were anaemia (haemoglobin = 91 g/L) and thrombocytopenia (platelets = 54 × 10⁹/L). Temperature would usually rise to around 39°C when a spike was noted, with fibrinogen at 1.7 g/L and aspartate aminotransferase (AST) at 194 U/L. An H-score of 220 was calculated following discussion with the specialist HLH team (Table 1).

**Table 1:** H-score calculation.

H-score component	Patient finding	Points
Known underlying immunosuppression	None known	0
Temperature	39°C	33
Organomegaly	None	0
Number of cytopenic lineages	2 (haemoglobin = 91 g/L; platelets = 54 × 10⁹/L)	24
Ferritin	>6,000 µg/L	50
Triglycerides	8.0 mmol/L	64
Fibrinogen	1.7 g/L	30
Aspartate aminotransferase	194 U/L	19
Haemophagocytosis on bone marrow	Not demonstrated	0
TOTAL	-	220

The HLH-2004 criteria were also later measured [4]. The patient had fever, two affected blood cell lineages, hypertriglyceridaemia, low fibrinogen, and marked hyperferritinaemia. Bone marrow aspirate did not demonstrate convincing haemophagocytosis, and there was no organomegaly. Flow cytometry reported no NK-cell activity identified and occasional CD25-positive cells. These observations should not be interpreted as a measured elevated soluble CD25 concentration. In view of this, the patient met 5/8 of the HLH-2004 criteria within the limits of the data available [4].

Whilst further investigations were ongoing, the patient self-discharged following notification that blood cultures taken approximately 24 hours earlier were positive for gram-positive cocci in clusters in both bottles. He was counselled about the risks but chose to leave hospital. He re-presented to the emergency department eight days later with fever and shortness of breath. On re-presentation, he received intravenous piperacillin-tazobactam and a stat dose of gentamicin. Repeat blood cultures obtained on readmission were negative.

Given the renal infarct and positive blood culture, infective endocarditis was considered an important differential diagnosis. Both transthoracic echocardiograms were negative for evidence of infective endocarditis. Transoesophageal echocardiography was discussed with cardiology but not performed. Although subsequent blood cultures were negative, an occult infective source could not be definitively excluded, and the positive result remained a possible clue to the HLH trigger.

The patient initially declined bone marrow aspiration but agreed during his second admission after further discussions supported by an interpretation service. The aspirate showed no evidence of haematological malignancy and no convincing haemophagocytosis, although slide quality was poor. Absence of demonstrable haemophagocytosis in this limited specimen does not rule out HLH.

Following specialist multidisciplinary review, intravenous methylprednisolone 1 g daily for three days and intravenous anakinra 200 mg twice daily were commenced. Fever and cytopenias subsequently improved. He transitioned to a tapering course of oral prednisolone and subcutaneous anakinra, with multidisciplinary outpatient follow-up and resolution of cytopenias reported. The temporal association with treatment is consistent with clinical improvement, yet the cause remains unidentified.

The timeline of the patient's clinical presentation, diagnostic evolution, and treatment is summarised in Table 2.

**Table 2:** Timeline of clinical presentation, diagnostic evolution, and treatment. MDT: multidisciplinary team; TTE: transthoracic echocardiography; HLH: haemophagocytic lymphohistiocytosis.

Time	Clinical/laboratory development	Clinical significance
Admission/Day 1	Acute right flank pain, vomiting, and polyuria; CT demonstrated right renal infarction	Initial diagnosis directed clinical management
Day 2	Persistent pyrexia despite antipyretics and antibiotics	Infection initially considered
Early admission	Repeated blood cultures initially negative; TTE ×2 without evidence of infective endocarditis	No clear infectious source identified
Progressive admission	Ferritin 6,345 → peak 28,904 µg/L; haemoglobin 94 g/L; platelets 41 ×10⁹/L; triglycerides 8.0 mmol/L	Emerging HLH phenotype
Approximately Day 12–14	HLH investigations initiated; H-score 220	93–96% probability of haemophagocytic syndrome
First admission	Patient self-discharged while diagnostic work-up remained ongoing	Further delay in diagnosis/treatment
Eight days later	Re-presented with persistent fever and dyspnoea	HLH work-up resumed
Second admission	Bone marrow aspiration performed; no evidence of malignancy	Malignancy trigger not identified
Following MDT review	IV methylprednisolone 1 g daily ×3 days + anakinra 200 mg twice daily	HLH-directed treatment
Subsequent follow-up	Clinical improvement and resolution of cytopenias; oral prednisolone and subcutaneous anakinra continued	Sustained response

## Discussion

This case demonstrates the diagnostic difficulty of adult HLH when a definite acute vascular abnormality initially provides a competing explanation for the presentation. The subsequent pattern of persistent fever, falling haemoglobin and platelet counts, hyperferritinaemia, and hypertriglyceridaemia warranted a broader differential diagnosis. The practical “three Fs” (fever, falling blood counts, and ferritin) may prompt earlier specialist discussion, while recognising that each feature is individually non-specific [2].

Published adult cohorts describe diagnostic delay and substantial mortality. One retrospective series reported a median 20-day delay to diagnosis [5,6]. Published case reports have also described HLH initially being managed as sepsis or another acute inflammatory illness before the emergence of cytopenias and marked hyperferritinaemia prompted reconsideration of the diagnosis [7,8].

Appropriate scoring systems for HLH have been established to help identify the probability of this diagnosis. The HLH-2004 diagnostic criteria provide a structured framework, while the H-score estimates the probability of reactive haemophagocytic syndrome [9]. More recent multicentre validation found mean sensitivity of 82.4% and specificity of 87.6% for an H-score threshold of 169 [10]. In this patient, the H-score of 220 provided strong support for the clinical diagnosis. The bone marrow sample showed no convincing haemophagocytosis and was of limited quality. However, other adult validation data have demonstrated variation in the optimal threshold according to the underlying clinical context, emphasising that scoring systems should support rather than replace clinical judgement [11]. The limitations and difficulties represented in this case reinforce the need to interpret diagnostic criteria alongside clinical judgement.

Ferritin is particularly useful as a screening marker but should not be interpreted in isolation. Marked hyperferritinaemia occurs in a number of conditions, including severe infection, malignancy, liver injury, and renal failure. In a study of adults with ferritin concentrations greater than 5,000 ng/mL, HLH represented only a minority of cases, demonstrating that severe hyperferritinaemia alone is insufficient to establish the diagnosis [12]. Consequently, the clinical trajectory and combination of findings are more informative than ferritin alone. In this case, the increase in ferritin from 6,345 to 28,904 µg/L occurred alongside a picture of worsening cytopenias and hypertriglyceridaemia, providing a more concerning pattern than an isolated ferritin elevation.

The concept of recognising the combination of fever, falling blood counts, and ferritin may therefore be particularly useful in clinical practice. In this case, the patient's persistent pyrexia was initially attributed to possible infection, and multiple antimicrobial regimens were administered without sustained resolution. Two transthoracic echocardiograms did not demonstrate infective endocarditis, while repeated microbiological investigations were initially negative. The persistence of fever despite antimicrobial therapy, together with the subsequent development of bi-cytopenia and marked hyperferritinaemia, eventually prompted reconsideration of HLH. Adult HLH expert recommendations recognise that the syndrome may be clinically indistinguishable from sepsis or multiple organ dysfunction and emphasise the importance of early recognition and multidisciplinary management [13].

The renal infarction represents an unusual aspect of this presentation. At admission, it directed attention towards thromboembolic, vascular, and inflammatory causes. Thrombophilia, antiphospholipid, and vasculitis testing were negative; ECG showed normal sinus rhythm, and transthoracic echocardiography did not reveal an embolic source or infective endocarditis. Haematology considered this likely an arterial event, but no clear cause was established. It is not possible to determine whether the infarction was independent of HLH or shared an underlying trigger, such as infection.

The positive blood cultures also merit consideration as a potential clue to an infectious trigger. Gram-positive cocci in clusters were identified in both bottles before the patient self-discharged. Following re-presentation, he was treated with intravenous piperacillin-tazobactam and a stat dose of gentamicin, and repeat blood cultures were negative. Two transthoracic echocardiograms did not show infective endocarditis, but transoesophageal imaging was not undertaken. Therefore, while no definitive HLH trigger was established, the possibility of an occult infection, including endocarditis, cannot be completely dismissed.

A systematic review and meta-analysis encompassing 18 cohorts and 3,570 adults reported pooled mortality of 49.5%, with mortality of 34.4% at 30 days and 57.3% at the longest reported follow-up [14]. These estimates refer to different follow-up windows and heterogeneous cohorts; the separate 31.0% estimate from a different retrospective study has been removed to avoid presenting non-comparable figures as equivalent. An observational analysis of 2,709 hospitalisations associated treatment initiation at or after day six with higher in-hospital mortality (OR: 2.00, 95% CI: 1.65-2.43), but cannot prove that treatment delay itself caused worse outcomes [15].

The England population-based study described an increase in age- and sex-adjusted HLH incidence from approximately one to two cases per million during 2000-2016, and approximately 0.8 cases per million among people aged 15-44 years [16]. These figures describe a different outcome from the mortality estimates and are kept distinct in Table 3.

**Table 3:** Incidence, effects, and prognosis in HLH. HLH: haemophagocytic lymphohistiocytosis.

Finding	Figure
HLH incidence in England.	~1-2 per million [16]
Incidence in ages 15-44 years.	0.8 per million [16]
Pooled adult HLH mortality	49.5% [14]
Pooled mortality by 30 days.	34.4.% [14]
Mortality at longest follow-up.	57.3% [14]
Increased mortality with delayed treatment.	OR = 2.00 [15]
H-score ≥ 169 diagnostic performance.	83.4% sensitivity and 87.6% specificity [10]

The patient’s initial reluctance to undergo serial sampling and marrow aspiration, together with a language barrier, complicated the diagnostic process. Use of an interpretation service supported shared decision-making and ultimately facilitated further investigation and outpatient treatment. This case cannot determine whether earlier HLH-directed treatment would have altered his course, but it illustrates the value of repeatedly reassessing a changing clinical picture and involving specialist teams early.

## Conclusions

Overall, this patient’s case highlights the importance of reconsidering the differential diagnosis when persistent fever and progressive haematological abnormalities are not explained by an initial acute diagnosis. The combination of fever, falling blood counts, and marked hyperferritinaemia should prompt consideration of HLH and early H-score calculation.

This case also demonstrates that diagnostic management is not solely dependent on laboratory testing. The patient's reluctance to undergo investigations and treatment, together with a language barrier, complicated the diagnostic process. The use of a language interpretation service facilitated subsequent discussions and improved engagement, ultimately allowing bone marrow examination and continued treatment. Effective communication and shared decision-making are therefore important components of managing complex cases of suspected HLH, particularly where prolonged investigation and potentially toxic immunomodulatory treatment are required. In this case, the renal infarct was considered likely arterial but remained unexplained despite negative vascular investigations. Although repeat blood cultures were negative and echocardiograms showed no infective endocarditis, the earlier positive culture leaves an infectious trigger uncertain. Bone marrow examination was non-diagnostic for haemophagocytosis. Early recognition, use of appropriate diagnostic criteria or scoring systems, specialist multidisciplinary input, and timely treatment are important, given the significant mortality associated with adult HLH.
